# The imbalance in the relationship between inflammatory and regulatory cytokines during gestational toxoplasmosis can be harmful to fetuses: A systematic review

**DOI:** 10.3389/fimmu.2023.1074760

**Published:** 2023-01-18

**Authors:** Priscilla Vilela dos Santos, Débora Nonato Miranda de Toledo, Débora Maria Soares de Souza, Tatiana Prata Menezes, Luiza Oliveira Perucci, Zolder Marinho Silva, Daniela Caldas Teixeira, Ed Wilson Rodrigues Vieira, Valter Ferreira de Andrade-Neto, Nathalia Sernizon Guimarães, André Talvani

**Affiliations:** ^1^ Laboratory of the Immunobiology of Inflammation, Department of Biological Sciences/Institute of Exact and Biological Sciences (ICEB), Federal University of Ouro Preto, Ouro Preto, MG, Brazil; ^2^ Graduate Program in Health and Nutrition, School of Nutrition, Federal University of Ouro Preto, Ouro Preto, MG, Brazil; ^3^ Faculty of Medical Sciences of Minas Gerais, Belo Horizonte, MG, Brazil; ^4^ Department of Maternal and Child Nursing and Public Health, Federal University of Minas Gerais, Belo Horizonte, MG, Brazil; ^5^ Laboratory of Malaria and Toxoplasmosis Biology, Department of Microbiology and Parasitology, Federal University of the Rio Grande do Norte, Natal, RN, Brazil; ^6^ Graduate Program of Health Science, Infectiology and Tropical Medicine, Federal University of Minas Gerais, Belo Horizonte, MG, Brazil

**Keywords:** *Toxoplasma gondii*, inflammation, cytokines, chemokines, gestation

## Abstract

**Objective:**

To evaluate the available information on inflammatory and regulatory plasma mediators in pregnant women (PW) diagnosed with toxoplasmosis. Source: The PubMed, Embase, Scopus, and Lilacs databases were evaluated until October 2022. Study eligibility criteria: This review was carried out following the PRISMA and registered on the PROSPERO platform (CRD42020203951). Studies that reported inflammatory mediators in PW with toxoplasmosis were considered.

**Evaluation methods:**

After excluding duplicate articles, two authors independently carried out the process of title and abstract exclusion, and a third resolved disagreements when necessary. The full text was evaluated to detect related articles. The extraction table was built from the following data: Author, year of publication, journal name and impact factors, country, study design, number of gestations and maternal age (years), gestational period, diagnosis of toxoplasmosis, levels of inflammatory markers, laboratory tests, and clinical significance. Methodological quality was assessed using Joanna Briggs Institute tools.

**Results:**

Of the 1,024 studies reported, only eight were included. Of the 868 PW included in this review, 20.2% were IgM+/IgG- and 50.8% were IgM-/IgG+ to *T. gondii*, and 29.0% uninfected. Infected PW presented higher plasma levels ofIL-5, IL-6, IL-8, IL-17, CCL5, and IL-10. Regarding the methodological quality, four studies obtained high quality. Data from this review pointed out the maintenance of the inflammatory pattern during pregnancy with a closely related to the parasite.

**Conclusion:**

Immune status in PW defined the course of the *T. gondii* infection, where the equilibrium between inflammatory and regulatory cytokines mitigated the harmful placenta and fetus effects.

**Systematic review registration:**

https://www.crd.york.ac.uk/prospero/, identifier CRD420203951.

## 1 Introduction

*Toxoplasma gondii* is a unicellular parasite capable of infecting mammals through the ingestion of mature cysts in contaminated soil or in meat, fruits, vegetables, untreated water, and unpasteurized milk (1–3). This protozoan is the cause of gestational toxoplasmosis (GT), an infection mainly evidenced in tropical climate countries where society is more exposed to mature oocysts in the environment in places with low investment in public health policies (4, 5). In this sense, an acronym TORCH has been widely used to highlight pathogens that can cause disturbances in fetal development and, in which placenta is the first and most important barrier to protect fetal tissues. TORCH is defined by *Toxoplasma gondii*, Other microorganisms (eg. coxsackievirus, syphilis virus, parvovirus B19, listeriosis, Zika virus, varicella-zoster virus, hepatitis virus, enterovirus, human immunodeficiency virus and the protozoan *Trypanosoma cruzi*), Rubella virus, Cytomegalovirus, and Herpes virus (6, 7).

Maternal serological screening during prenatal care allows the identification of infected and susceptible pregnant women for follow-up to prevent fetal infection by *T. gondii*. This follow-up supports the diagnosis of congenital toxoplasmosis (CT), which is based mainly on indirect methods, such as serology, but also on methods of direct detection of the parasite (8). Most CT cases are asymptomatic. However, *T. gondii* infection can lead to abortion and several syndromes involving neurological and neurocognitive deficits and chorioretinitis in fetuses (9–11).

*T. gondii* modulates the immune system of the infected host to establish a continuous infection and thus maintain a replicative niche. This process occurs through the regulation of host gene transcription, leading to an imbalance of inflammatory signaling pathways that result in leukocyte migration and modulation of their adhesion to the endothelium, release of immunoregulatory cytokines, and cell apoptosis (12–14). Immune cells infected with *T. gondii* tachyzoite cause activation of cells of the innate immune response, generating inflammatory cytokines and triggering the adaptive immune response (15). Inflammatory cytokines amplify the destruction of tryptophan in fibroblasts, preventing parasite replication (16).

Chemokines (eg. CCL2 and CXCL2) also contribute to this amplification of the immune response against *T. gondii*. They promote migration of Ly6C^high^ CCR2+ monocytes and neutrophils phenotypes to the focus of infection and, increase expression/production of IFN-γ and neutrophil extracellular traps amplifying the innate and adaptive immune responses (17–20). However, Ly6C^high^ CCR2+ monocytes also produce IL-10, which exert a suppression effect on *T. gondii*, in particular in *Toxoplasma* encephalitis (21). In non-infection conditions, the Foxp3+ CD4+ Treg cells are important sources of IL-10 and, during *T. gondii* infection, these cells are tightly controlled. However, these cells are always present to contribute to the immunosuppression to avoid excessive immunopathology, maintain maternal-fetal tolerance and, to promote fetal development during gestation (22). Therefore, not only IL-10, but also IL-22, act as regulatory cytokines suppressing part of *T. gondii*-induced inflammation, while IL-33 exerts dual pro-inflammatory and regulatory functions. IL-33 can improve CCL2 expression with recruitment of CCR2+ monocytes, but it can amplify Th2 response with production of IL-10 and IL-22 by M2 macrophages and Th-17-cells, respectively which, in turn, activates Treg cells (23–25).

Normal pregnancy is described by the balance between inflammatory and regulatory immune responses. Indeed, its success requires an increased number of regulatory T cells (Tregs) compared to the nonpregnant state, which is essential for maternal–fetal tolerance (26). These cells are the main sources of TGF-β, another cytokine that regulates immune responses against *T. gondii* through mechanisms such as the development of Tregs and clonal expansion of Th17 lymphocytes, which are essential factors in the immune response against the parasite (27). Whether Tregs are compromised during pregnancy, the possibilities of complications are greater (28). However, infectious processes concomitant with pregnancy can modify this homeostasis and thus affect fetal development (28, 29).

The distribution of *T. gondii* genotypes varies in different geographic areas around the world, which comprises around 950 isolates with 15 haplogroups capable to define 6 major clades (30). In these haplogroups, 3 main clonal lineages were identified, distributed in North America and Europe, with distinct atypical genotypes and new clonal lineage found in South America. The hallmark behind these studies is the conception that distinct and atypical genotypes dictate the transmissibility and pathogenicity (31, 32). In this context, a particular attention must be given to those cases where the maternal protection can be breached during pregnancy, in human or in experimental models, due to different genotypes (33, 34) or due to the immune molecular compounds (35).

Considering that the regulation of the maternal immune system during GT is not well understood, this systematic review aimed to explore the profile of proinflammatory and regulatory mediators in pregnant women with toxoplasmosis described in the literature.

## 2 Methods

### 2.1 Protocol and registration

This systematic review was conducted according to the PRISMA [Preferred Reporting Items for Systematic Review and Meta-analysis] guidelines (36) and to a recently submitted register linked to PROSPERO, under this identification CRD420203951.

### 2.2 Search strategy and selection criteria

Studies that reported the profile of proinflammatory and regulatory mediators in pregnant women with toxoplasmosis were considered. We conducted a comprehensive systematic search in PubMed, Embase, Scopus, and Lilacs. No limit on the year of publication was set, and the final search was updated until October 2022 without language restrictions. Additionally, the reference lists of the articles retrieved and related review studies were also hand-screened to find eligible trials that might have been missed.

Through the acronym PECO, the definition of the key words was carried out through a search using Medical Subject Headings (MeSH) and was adapted for each database using DeCS (LILACS) and Emtree (EMBASE) descriptors (**Supplementary Material**). Retrieved titles and abstracts were reviewed separately by two authors to find related studies.

### 2.3 Study selection

After excluding duplicate articles, two independently authors carried out the process of title and abstract exclusion, and a third resolved disagreements when necessary. The full text was evaluated to detect related articles. Finally, original articles were included in the present systematic review if they had the following criteria: 1) cross-sectional cohort or case-control designs; 2) studies that evaluated pregnant women infected with *T. gondii;* and 3) publications that reported sufficient data on the *T. gondii* immune response of pregnant women.

Duplicated studies or with unclear information and in which we did not receive any feedback from the corresponding author(s) after email, studies carried out in nonpregnant women, parturient, research that evaluated anti-inflammatories, vaccines, or other medications, research carried out with pregnant women with other basic infections and narrative, integrative, systematic reviews, and meta-analyses, were excluded.

### 2.4 Data extraction

Two independent authors extracted information from the primary studies reviewed, and an additional reviewer resolved disagreements. The following information was extracted: author, year of publication, journal name and impact factor, country, study design, number of gestations, mean maternal age (years), gestational period, diagnosis of toxoplasmosis, mean levels/expression of proinflammatory (IFN-γ, IL-1α, IL-1β, IL-2, IL-5, IL-6, IL-8, IL-12, IL-17, IL-17A, IL-33, CCL2, CCL4, CCL5, CCL7, CXCL1) and regulatory (TGF-β, IL-4, IL-10) biomarkers, type of laboratory tests performed, and the clinical significance of the study.

### 2.5 Quality assessment

The Joanna Brigges Institute Tools (37) were used by two reviewers to identify potential risks of bias. Any disagreements in the data extraction and the risk of bias assessment were resolved by a third reviewer. This tool measures the probable causes of bias in cross-sectional, cohort, and case-control studies, which include: (i) measurement of exposure in a valid and reliable way, (ii) sample in line with the study design, (iii) identification of confounding factors, (iv) validation of study quality, (v) adequate statistical analysis, and (vi) incomplete results data. The responses to the questionnaires could be classified as: “yes”, “no”, “unclear” or “not applicable”. Based on the recommendations of this tool, a judgment of each domain was recorded as “high”, “moderate”, “low”, or “very low” risk of bias.

## 3 Results

Our research retrieved 1,024 studies, of which 28 were duplicates. Most studies were discarded after title and abstract analysis (n = 996) and 56 were selected for full-text screening. In total, eight studies were included in our review. The flowchart of eligibility is presented in the **Supplementary Material**, and the synthesis of the eight selected studies is presented in **Table 1**.

**Table 1:** Description of the studies included in the systematic review.

Authors and year	Journal (I.F.)	Geographical locality	Study design	Pregnant women	Trimester of the pregnancy
(38)	Gastroenterology and Hepatology (3.437)	Teerã, Iran	Cross-sectional	N=268 AR: N. I.	UN
(39)	Journal Obstetrics Gynaecology (6.120)	Taiwan, China	Cross-sectional	N=220 AR: N.I.	UN
(40)	The Journal of Infectious Diseases (5.186)	Cali, Colombia, USA	Cohort	**USA** N=144 AR:16-47 years AA: 29,3 years **Colombia** N=113 AR: 16-46 years AA: 28,3 years	UN
(41)	Egyptian Journal of Human Medical Genetics (0.440)	Giza, Egypt	Control case	N=61 AR: 27-36 years AA: 31.5 years	UN
(42)	Frontiers in Immunology (6.429)	City of Mexico, Mexico	Cross-sectional	N=11 AR: 18-38 years AA: 28 years	1^st^ trimester (n=6); 2^nd^ trimester (n=3); 3^rd^ trimester (n=2)
(43)	Journal of Parasite Research (1.872)	Sudan, India	Cross-sectional	N=68 AA: 30.28 years	UN
(44)	Frontiers in Immunology (6.429)	City of Mexico, Mexico	Cross-sectional	N=17 AR: N. I. AA: 29 years	1^st^ trimester (n=6); 2^nd^ trimester (n=3); 3^rd^ trimester (n=8)
(25)	Acta Tropica (2.555)	Santa Cruz, Brasil	Cross-sectional	N=101 AA: 30.5 years	UN

IF, impact factor; UN, uninformed; N, number; AR, age range; AA, age average.

### 3.1 Characteristics of the study

The selected studies were published between 2011 and 2020, with the majority dating from the last five years. They were published in journals with an impact factor between 0.44 and 6.42, with half of them having an impact factor ≥ 5.1. Only one study was published in the predatory journal name (43). The eligible articles were conducted in China (39), Iran (38), Colombia, in collaboration with the United States (40), India (43), Egypt (41), Mexico (42, 44) and Brazil (25).

Six of the included articles had a cross-sectional design (75%), one cohort (12.5%), and one was a case-control study (12.5%). The number of participants in each study ranged from 11 to 300 pregnant women with a mean age of 29.8 years. Regarding the clinical stages of the pathology evaluated, 197 (20.2%) pregnant women were IgM+, 496 (50.8%) were IgG+, and 283 (29.0%) had no previous exposure to the parasite. Only two articles reported the administration of drugs to control toxoplasmosis infection, this being performed after the collection of the biological material analyzed.

### 3.2 Inflammatory mediators in the GT

We evaluated the mean levels of plasma inflammatory and regulatory cytokine levels in the data obtained from these studies (**Tables 2**, **3**). In comparison with pregnant women without *T. gondii* infection, those with chronic toxoplasmosis (IgG+/IgGM-) presented increased levels of IFN-γ, IL-1β, IL-2, IL-4, IL-5, IL-6, IL-8, IL-12, and IL-17 and lower levels of the chemokine CCL4. It was also observed that pregnant women in the acute phase of toxoplasmosis (IgM+/IgG-) presented higher levels of TNF, IL-1β, IL-2, and IL-12. When the IgM+/IgG- pregnant women were compared with those with IgM-/IgG+, higher plasma levels of CCL7, TNF, lymphotoxin alpha, and IFN-γ were highlighted in parallel with decreased levels of CCL4 and unaltered plasma levels of CCL2, IL-17A, and IL-33.

**Table 2:** Inflammatory mediators in *T. gondii*-infected pregnant women .

Authors, year	Diagnostic of toxoplasmosis (IgG/IgM)	Inflammatory mediators	Clinical meaning
(38)	IgM+ (N= 66) IgG+ (N= 202)	IL-8	PWGPWH: ↑ IL-8
(39)	IgM+ low avidness (N= 6) IgG+ (N= 20) IgG- (N=194)	IFN-γ, IL-1β, IL-2, IL-5, IL-8, IL-6, IL-12, TNF, TNF-β	PWMPWH: ↑ IL-1β, IL-2, IL-12; ↔ IL-5, IL-6, IL-8; ↓ TNF-β PWGPWH: ↑ IFN-γ, IL-1β, IL-2, IL-4, IL-5, IL-6, IL-8, IL-12
(40)	**USA:** IgG and IgM- (N=48) IgM+ (N=47) IgG+ (N=49) **Colombia:** IgM+ (N=13) IgG+ (N=50)	IFN-γ, TNF, lymphotoxin-α IL1-α, IL-2, IL-4, IL-8, IL-17, CXCL1, CCL4, CCL5, CCL7	PWMPWG: ↑ CCL7, TNF-β; ↓ CCL4
(41)	IgM+ (N=26) IgG+ (N=35)	TNF, IFN-γ	PWMPWG: ↑ TNF, IFN-γ
(42)	IgM+ (N=7) IgG+ (N=10) IgG- (N=1)	IFN-γ	CTNT: ↔ IFN-γ
(43)	IgM+ (N=3) IgG+ (N=65)	IL-8, IL-17	PWGPWH: ↑ IL-8, IL-17
(25)	IgM+ (N=26) IgG+ low avidness (N=3) IgG+ high avidness (N=109)	IL-17A, IL-33, CCL2	PWMPWG: ↔ CCL2, IL-33, IL-17A
(44)	IgM+ (N=12) IgG+ low avidness (n=4) IgG- low avidness (n=1)	TNF, IFN-γ, IL-6	TPWNTPW: ↑ TNF, IFN-γ, IL-6

IgG, immunoglobulin G; IgM, immunoglobulin M; PWGPWH, IgG + pregnant woman versus healthy pregnant woman; PWMPWH, IgM + pregnant woman versus healthy pregnant woman; PWMPWG, IgM + pregnant woman versus IgG+ pregnant woman; TPWNTPW, transmitting pregnant woman versus not transmitting pregnant woman; CTNT, congenital transmission versus non-transmission.

**Table 3:** Plasma regulatory mediators in *T. gondii*-infected pregnant women.

Authors, year	Diagnostic of toxoplasmosis (IgG/IgM)	Regulatory mediators	Clinical analysis
(39)	IgM+ (N= 6) IgG+ (N= 20)	IL-4, IL-10	PWMPWH: ↑ IL-10; ↔ IL-4
(40)	**USA:** IgG e IgM- (N=48) IgM+ (N=47) IgG+ (N=49) **Colombia:** IgM+ (N=13) IgG+ (N=50)	TGF-β	PWMPWG: ↔ TGF-β
(41)	IgM+ (N=26) IgG+ (N=35)	IL-10	PWMPWG: ↔ IL-10
(42)	IgM+ (N=7) IgG+ (N=10) IgG- (N=1)	TGF-β	CTNT: ↓ TGF-β

IgG, immunoglobulin G; IgM, immunoglobulin M; PWMPWH, IgM + pregnant woman versus healthy pregnant woman; PWMPWG, IgM + pregnant woman versus IgG+ pregnant woman; CTNT, congenital transmission versus non-transmission.

### 3.3 Quality of evidence

From the eight surveys evaluated, four studies were classified as having high quality of evidence and, the four studies presented medium quality of evidence, according to Joanna Brigges Institute tools (**Supplementary Material**).

## 4 Discussion

Each uninfected stage of pregnancy needs a specific immune response: (i) the first trimester requires an environment of inflammation; (ii) the second trimester is characterized by a regulatory pattern, where fetal development and growth are priorities and; (ii) finally, in the last trimester, the placental tissue requires a resolution of this inflammation necessary to promote uterine contractions and the release of the fetus and the placenta from the mother´s body (45). However, when the placental tissue is infected by *T. gondii*, it can disrupt this gestational homeostasis and induce a clinical condition akin to a fetal inflammatory response syndrome, in which the placental environment releases high levels of IL-1, IL-6, IL-8, IL-17, and TNF to act on this parasite contention (38–41, 43, 44).

This syndrome caused by *T. gondii* is responsible for the systemic activation of the fetal and maternal immune system (42). Distinct biomarkers work in a Th1/Th2/Th17 balance thought cytokines (IFN-g, TNF, TGF-beta, IL-1beta, Il-10, IL-12, IL-17, IL-18, IL-22, IL-33, etc), chemokines/receptors (CCL2, CCL22, CXCL1, CXCL2, CXCR3, etc), as well as by Toll-like receptors, nucleotide-binding oligomerization domain-like receptors, and inflammasomes (eg.NLRP3, NLRP5 and NAIP1), defining the pathogenesis behind *T. gondii* infection during pregnancy (19, 24, 25, 27, 35, 38, 46–48). Beyond this, it is also remarkable to highlight the importance of the genetic variability of *T. gondii* identified in different continents/countries and, how variability can dictate the prognostic of gestation (30–32 *)*. In this case, in a multifactorial way, atypical characteristics may originate in specific genotypes/phenotypes of the parasite, the host, or both.

Supported by these differences in host/parasites genotypes, human and experimental models in *T. gondii* infection have gain strength in the last decades. Experimental models allow that scientists control variables and go deeper in terms of immunopathology and genetic background in mammalians and parasites. Parasites that were, isolated from human patients are, usually, inoculated in rodents to understand the chronology, biochemical, molecular, morphological and immunological in acute and chronic (eg. congenital and ocular) toxoplasmosis (49). These variables would find a hindrance to be controlled and understand in humans. The cell culture and, the 2D and 3D cell cultures are fundamental models to understand *T. gondii* biology (biochemical and molecular), its virulence and replication (50). To investigate *T. gondii* biology, transmission and immunopathology, animals have been used such as porks, cats, monkeys (51–53), but the large number of studies are focused on mice. Mice possess a good response to neurological, behavior, congenital and ocular disturbances when compared with other mammalians and, better cost-effective in terms of space and reproduction in laboratory. However, it is fundamental to comprehend that strains of *T. gondii* dictates its virulence and, the high virulent Type I strains are usually lethal at all doses to all laboratory mice lineages, whereas types II and III strains induce stronger inflammatory response and, then, are less pathogenic to these rodents (54). To investigate placental transmission, the fetus safety and the maternal immune response, a cross mash between clinical and experimental models (cell cultures and animals) are very positive to improve knowledge and instigate potential new strategies such as chemotherapy and vaccines targets or even the understand of the clinical concerning *T. gondii* infection in homeostatic body or in an immunosuppressed condition.

*T. gondii* drives a classical Th1 response in an immunocompetent host leading a production of pro-inflammatory mediators, in which IFN-γ, TNF, IL-12, IL-18, IL-1β, nitric oxide, CCL2, CXCL2, CXCR3 and MyD88-dependenty factors, are critical for host survival (17, 55, 56). Indeed, this protective scenario is mediated by antigen-presenting cells (eg. dendritic cells and macrophages), that activate T cells to intensify the development of Th1 cells and antigen-specific killer CD8 T cells. The activated macrophages/dendritic cells, through Tool-like receptors and C5a/C5aR1 axis, are able to produce IL-1α, IL-1β, IL-12 and TNF that triggers the proliferation of T CD4+, CD8+ and NK cells, mediating the cytotoxicity against parasites and increasing the IFN-γ production (57–59). Indirect effects promoted by these inflammatory mediators lead susceptibility or resistance in human and animals’ immunocompetent hosts are dependent on the evolutive form of the parasite and on the parasite strain. For example, the RH strain of *T. gondii* (Type I or more virulent strains) induces less translocation of NK-kB which, in turn, results in production of regulatory mediators (IL-10, IL-27 and TGFB1) and contributes to the proliferation of the *parasites* (60). On the other hand, the ME49 and DEG strains belong to Type II strain of *T. gondii* (less virulent) and promote translocation of NF-_k_B from the cytoplasm to the nucleus of distinct activated cells such as splenocytes, B-cells, macrophages which, in turn, enhance the production of pro-inflammatory cytokines IL-1α, IL-12, IL-18, IFN-γ, TNF, others and kills parasite with an intensification of tissue damage (61). Indeed, a balance among Th1, Th2 and Th17 immune responses would be desirable to stablish a resistance against *T. gondii* invasion with less pathology development to the hosts.

The pathways leading to the production of IL-1β in human cells during *T. gondii* infection are not well understood, as are the inflammatory roles of IFN-γ in GT. Gov et al. (62) described through an *in vitro* study that *T. gondii* is able to promote IL-1β transcript, processing and release in human monocytic cell line. Based on that, IL-1β was proposed as a key regulator of the innate inflammatory response against *T. gondii* in a close-dependency of the parasite protein (GRA15) and inflammasome components ASC and caspase-1. This study highlighted the importance of IL-1β in controlling *T. gondii*, but also highlighted that this role needs to be regulated to avoid damage to the infected hosts. In this sense, IFN-γ and TNF also elevated in women with GT to control *T. gondii* in the initial phase of infection, but partial regulation is also crucial to ensure fetus protection (46).

The chemokine IL-8 (CXCL8) is also associated with a higher risk of CT (40, 43). Placental and intestinal epithelial cell susceptibility to *T. gondii* is mediated by the host immune response, and this parasite elicits IL-8 secretion (63). IL-8 is responsible for the activation and recirculation of neutrophils, and these cells can phagocytose and eliminate Toxoplasma tachyzoites. Therefore, a higher concentration of plasmatic IL-8 could be related to a higher risk of CT due to the presence of a high load of parasites in the mother´s body and, consequently, in the placental environment.

In parallel with this inflammatory pattern related to CT, *T. gondii*-infected women also produce high plasma levels of regulatory IL-10 (39). IL-10 can deactivate macrophages and facilitate the intracellular survival of parasites. However, this partial immunosuppression or an equilibrium concerning inflammatory and regulatory cytokines can benefit both *T. gondii* and pregnant mammalian hosts, avoiding abortion (64, 65).

The proposal of potential inflammatory markers during GT is desirable as a medical tool to predict the course of pregnancy in *T. gondii*-infected women. In this sense, low levels of plasma TGF-β were detected in infected mothers with parasite transplacental infection (42). TGF-β may act as a regulator or protective marker during GT and, at low levels, lower the local defense against *T. gondii*, which can increase the risk of abortion (66, 67).

Regarding the analysis of inflammatory mediators, four studies were performed using the enzyme-linked immunosorbent assay (ELISA) method and, in the Chou et al. (39) and Pernas et al. (40) studies, the multiplex ELISA was applied. In the Gómez-Chávez et al. (42) and Gómez-Chávez et al. (44) studies, the flow cytometry technique was applied for the quantification of cytokines. In parallel with these cytokine analyses, all authors also described how the seroprevalence of *T. gondii* was performed. In all studies, the ELISA diagnostic method was used, and in the study conducted by Gómez-Chávez et al. (42), western blotting was also performed. Furthermore, in some cases, the avidity test was also applied to certify *T. gondii* acute infection, in which a high result for IgG avidity meant less risk for the fetus (25, 39, 44).

In summary, this systematic review highlighted the existence of an attenuating relationship between the balance of inflammatory and regulatory cytokines during GT and how an imbalance in this production can be harmful to the fetus, particularly in the first two trimesters of pregnancy. Further studies should be performed to examine the genetic background of the women, the quantitative levels of inflammatory mediators, and consider the genetic background of the parasites involved in the GT. The identification of potential inflammatory markers is urgently needed in the obstetrics field for the follow-up and prognosis of pregnancy in *T. gondii*-infected women. However, it is healthy to consider that inflammatory and regulatory biomarkers are both elements that control or exacerbate the *T. gondii* pathogenesis in a Taoism conception of the “Ying and Yang” whereas biomarkers act as essential keys to eliminate parasites and maintain the equilibrium of the maternal health during fetus development. Despite this dynamic scenario of eliminate parasites with controlled immunopathogenesis, the key-points are comprehended how different biomarkers work response to the variable genetic background of hosts and parasites and, propose clinical interventions to protect and/or improve the maternal-fetus relationship.

The absence of information about the gestational period and the body mass index of the participants were important limiting factors of this study. This fact makes it impossible to consider the nutritional status of these pregnant women, since obesity or excess weight can promote an increase in the systemic inflammatory state, which can interfere in the inflammatory context during TG. Another limiting factor observed was the lack of data regarding pharmacological treatment to control the disease, considering that the late start of medication can promote severe damage to the fetus.

## Author contributions

Conceptualization and/or supervision: AT, NS, VFA-N, PS, DNT. Writing original draft preparation: PS, DNT, NS, VFA-N, AT. Data curation and resource: PS, DNT, DS, TM, LP, ZS, DCT, EV. All authors contributed to the article and approved the submitted version.
