# Supplementary material for: The imbalance in the relationship between inflammatory and regulatory cytokines during gestational toxoplasmosis can be harmful to fetuses: A systematic review
Source: Front Immunol. 2023 Jan 18;14:1074760. doi: 10.3389/fimmu.2023.1074760 (PMC9889920; doi:10.3389/fimmu.2023.1074760)
Supplement: Supplementary file 1 [file DataSheet_1.docx]

**Supplementary Material**

**Search Strategy**

Toxoplasmosis [Mesh] OR *Toxoplasma gondii* Infection OR Infection, *Toxoplasma gondii* OR Toxoplasmosis, Congenital [Mesh] OR Toxoplasma Infections, Congenital OR Congenital Infection, *Toxoplasma gondii* OR Congenital Toxoplasma Infections OR Congenital Toxoplasma Infection OR Infection, Congenital Toxoplasma OR Infections, Congenital Toxoplasma OR Toxoplasma Infection, Congenital OR Congenital Toxoplasmosis OR Congenital Toxoplasmosis OR Toxoplasmosis, Congenital OR Congenital *Toxoplasma gondii* Infection OR Toxoplasmosis, Fetal OR Fetal Toxoplasmosis OR Fetal Toxoplasmosis OR Toxoplasmosis, Fetal OR Toxoplasmosis, Prenatal OR Prenatal Toxoplasmosis OR Prenatal Toxoplasmosis OR Toxoplasmosis, Prenatal. Interleukin-12 [Mesh] OR Natural Killer Cell Stimulatory Factor OR IL-12 OR Cytotoxic Lymphocyte Maturation Factor OR IL 12 OR IL-12 p70 OR Interleukin-12 p70 OR Interleukin 12 p70 OR Interleukin 12 OR IL12 OR Edodekin Alfa OR Interleukin-17 [Mesh] OR Interleukin 17 OR CTLA-8 OR IL-17 OR Interleukin-17F OR Interleukin 17F OR IL-17F OR Cytokine ML-1 OR Cytokine ML 1 OR Interleukin-17C OR Interleukin 17C OR IL-17C OR Cytokine CX2 OR CX2, Cytokine OR Interleukin-17E OR Interleukin 17E OR Interleukin-25 OR Interleukin 25 OR IL-17E OR IL 17E OR Interleukin-17A OR Interleukin 17A OR Cytotoxic T lymphocyte-Associated Antigen 8 OR Cytotoxic T lymphocyte Associated Antigen 8 OR IL-17A OR CTLA8 OR Interleukin-17B OR Interleukin 17B OR IL-17B OR IL32 protein, human [Mesh] OR IL-32, human OR NK4 protein, human OR interleukin 32 protein, human OR Interleukin-32, human OR natural killer cells protein 4, human OR natural killer cell transcript 4 protein, human OR Interleukin-6 [Mesh] OR Interleukin 6 OR IL6 OR B-Cell Stimulatory Factor 2 OR B-Cell Stimulatory Factor-2 OR Differentiation Factor-2, B-Cell OR Differentiation Factor 2, B Cell OR B-Cell Differentiation Factor-2 OR B Cell Differentiation Factor 2 OR BSF-2 OR Hybridoma Growth Factor OR Growth Factor, Hybridoma OR IFN-beta 2 OR Plasmacytoma Growth Factor OR Growth Factor, Plasmacytoma OR Hepatocyte-Stimulating Factor OR Hepatocyte Stimulating Factor OR MGI-2 OR Myeloid Differentiation-Inducing Protein OR Differentiation-Inducing Protein, Myeloid OR Myeloid Differentiation Inducing Protein OR B-Cell Differentiation Factor OR B Cell Differentiation Factor OR Differentiation Factor, B-Cell OR Differentiation Factor, B Cell OR IL-6 OR Interferon beta-2 OR Interferon beta 2 OR beta-2, Interferon OR B Cell Stimulatory Factor-2 OR B Cell Stimulatory Factor 2 OR Interleukin-1 [Mesh] OR Interleukin 1 OR IL-1 OR T Helper Factor OR Lymphocyte-Activating Factor OR Lymphocyte Activating Factor OR Macrophage Cell Factor OR Epidermal Cell Derived Thymocyte-Activating Factor OR Epidermal Cell Derived Thymocyte Activating Factor OR Interleukin I OR Interferon-gamma [Mesh] OR gamma-Interferon OR Interferon, Immune OR Immune Interferon OR Type II Interferon OR Interferon, Type II OR Interferon Type II OR Interferon, gamma OR Tumor Necrosis Factor-alpha [Mesh] OR Tumor Necrosis Factor alpha OR Cachectin OR Cachectin-Tumor Necrosis Factor OR Cachectin Tumor Necrosis Factor OR Tumor Necrosis Factor Ligand Superfamily Member 2 OR Tumor Necrosis Factor OR TNF Superfamily, Member 2 OR TNFalpha OR TNF-alpha OR Chemokines [Mesh] OR Cytokines, Chemotactic OR Intercrines OR Chemotactic Cytokines OR resolvin D1 [Mesh] OR 7,8,17-trihydroxy-4,9,11,13,15,19-docosahexaenoic acid OR RvD1 fatty acid OR resolvin D2 [Mesh] OR (4Z,7R,8E,10Z,12E,14E,17S,19Z)-7,16,17-trihydroxy-4,8,10,12,14,19-docosahexaenoic acid OR RvD2 compound OR 13,14-dihydroxydocosahexaenoic acid [Mesh] OR 13R,14S-diHDHA OR maresin 2 OR 7,14-dihydroxydocosa-4,8,10,12,16,19-hexaenoic acid [Mesh] OR MaR1 compound OR maresin 1 OR Mphi mediator in resolving inflammation OR lipoxin B4 [Mesh] OR 5D,14,15L-trihydroxy-6,8,10,12-eicosatetraenoic acid OR LXB4 OR 5,14,15-trihydroxy-6,8,10,12-eicosatetraenoic acid OR 5,14,15-THET OR 5-methyl-LXB4 OR “15-epi-LXA4” OR “LXA4” OR protectin [Mesh] OR neuroprotectin D1 OR 10R,17S-dihydroxy-docosa-4Z,7Z,11E,13E,15Z,19Z-hexaenoic acid OR 10,17-dihydroxydocosa-4,7,11,13,15,19-hexaenoic acid [Mesh] OR protectin DX.resolvinD1 [Supplementary Concept]] OR resolvinD2 [Supplementary Concept] OR 13,14-dihydroxydocosahexaenoic acid [Supplementary Concept] OR 7,14-dihydroxydocosa-4,8,10,12,16,19-hexaenoic acid [Supplementary Concept] OR lipoxin B4 [Supplementary Concept] OR protectin [Supplementary Concept] OR 10,17-dihydroxydocosa-4,7,11,13,15,19-hexaenoic acid [Supplementary Concept] OR Interleukin-12 [Mesh] OR Interleukin-17 [Mesh] OR IL32 protein, human [Mesh] OR Interleukin-6 [Mesh] OR Interleukin-1 [Mesh] OR Interferon-gamma [Mesh] OR Tumor Necrosis Factor-alpha [Mesh] OR Chemokines [Mesh].

**Supplementary Material - Quality assessment of evidence**

| **Author** | **1** | **2** | **3** | **4** | **5** | **6** | **7** | **8** | **Overall** |
| --- | --- | --- | --- | --- | --- | --- | --- | --- | --- |
| Chou et al, 2011 | UN | Y | Y | Y | N | NA | Y | Y | Moderate Quality Review |
| Gómez-Chavez et al, 2019 | Y | Y | Y | Y | N | NA | Y | Y | High Quality Review |
| Andrade et al, 2020 | Y | Y | Y | Y | N | NA | Y | Y | High Quality Review |
| Rostami Nejad et al, 2011 | Y | UN | Y | Y | N | NA | Y | Y | Moderate Quality Review |
| Gómez-Chavez et al, 2020 | Y | Y | Y | Y | N | NA | Y | Y | High Quality Review |
| Wahaj et al., 2019 | UN | Y | Y | Y | N | NA | Y | Y | Moderate Quality Review |

**Quality assessment of cross-sectional studies by JBI scale.**

| *Note: 1. Were patient’s demographic characteristics clearly described?* |
| --- |
| *2. Was the patient’s history clearly described and presented as a timeline?* |
| *3. Was the current clinical condition of the patient on presentation clearly described?* |
| *4. Were diagnostic tests or assessment methods and the results clearly described?* |
| *5. Was the intervention(s) or treatment procedure(s) clearly described?* |
| *6. Was the post-intervention clinical condition clearly described?* |
| *7. Were adverse events (harms) or unanticipated events identified and described?* |
| *8. Does the case report provide takeaway lessons?* |

**Quality assessment of cohort study by JBI scale.**

| **Author** | | **1** | **2** | **3** | **4** | **5** | **6** | **7** | **8** | **9** | **10** | **11** | **12** | **Overall** | |
| --- | --- | --- | --- | --- | --- | --- | --- | --- | --- | --- | --- | --- | --- | --- | --- |
| Pernas et al, 2014 | | N | Y | Y | Y | N | NA | Y | Y | UN | Y | NA | Y | Moderate Quality Review | |
| *Note: 1. Were the two groups similar and recruited from the same population?* | | | | | | | | | | | | | |  |  |
| *2. Were the exposures measured similarly to assign people* | | | | | | | | | | | | | |  |  |
| *3. to both exposed and unexposed groups?* | | | | | | | | | | | | | |  |  |
| *4. Was the exposure measured in a valid and reliable way?* | | | | | | | | | | | | | |  |  |
| *5. Were confounding factors identified?* | | | | | | | | | | | | | |  |  |
| *6. Were strategies to deal with confounding factors stated?* | | | | | | | | | | | | | |  |  |
| *7. Were the groups/participants free of the outcome at the start of the study (or at the moment of exposure)?* | | | | | | | | | | | | | |  |  |
| *8. Were the outcomes measured in a valid and reliable way?* | | | | | | | | | | | | | |  |  |
| *9. Was the follow up time reported and sufficient to be long enough for outcomes to occur?* | | | | | | | | | | | | | |  |  |
| *10. Was follow up complete, and if not, were the reasons to loss to follow up described and explored?* | | | | | | | | | | | | | |  |  |
| *11. Were strategies to address incomplete follow up utilized?* | | | | | | | | | | | | | |  |  |
| *12. Was appropriate statistical analysis used?* | | | | | | | | | | | | | |  |  |

**Supplementary Material - Quality assessment of cohort study by JBI scale.**

| **Author** | | **1** | **2** | **3** | **4** | **5** | **6** | **7** | **8** | **9** | **10** | **Overall** | |
| --- | --- | --- | --- | --- | --- | --- | --- | --- | --- | --- | --- | --- | --- |
| El-Sherbini *et al*, 2019 | | Y | Y | Y | Y | Y | N | NA | Y | Y | Y | High Quality Review | |
| *Note: 1. Were the groups comparable other than the presence of disease in cases or the absence of disease in controls?* | | | | | | | | | | | |  |  |
| *2. Were cases and controls matched appropriately?* | | | | | | | | | | | |  |  |
| *3. Were the same criteria used for identification of cases and controls?* | | | | | | | | | | | |  |  |
| *4. Was exposure measured in a standard, valid and reliable way?* | | | | | | | | | | | |  |  |
| *5. Was exposure measured in the same way for cases and controls?* | | | | | | | | | | | |  |  |
| *6. Were confounding factors identified?* | | | | | | | | | | | |  |  |
| *7. Were strategies to deal with confounding factors stated?* | | | | | | | | | | | |  |  |
| *8. Were outcomes assessed in a standard, valid and reliable way for cases and controls?* | | | | | | | | | | | |  |  |
| *9. Was the exposure period of interest long enough to be meaningful?* | | | | | | | | | | | |  |  |
| *10. Was appropriate statistical analysis used?* | | | | | | | | | | | |  |  |
